# Early prelingual auditory and language development in children with simultaneous bilateral and unilateral cochlear implants

**DOI:** 10.3389/fped.2022.999689

**Published:** 2022-11-03

**Authors:** Xiaoling Yin, Hailing Gu, Weili Kong, Gang Li, Yun Zheng

**Affiliations:** Department of Otolaryngology/Head and Neck Surgery, Hearing Center/Hearing & Speech Science Laboratory, West China Hospital of Sichuan University, Chengdu, China

**Keywords:** cochlear implant, simultaneous bilateral implantation, mandarin-speaking children, early prelingual auditory development, early language development

## Abstract

**Purpose:**

This current study aimed to explore early prelingual auditory development (EPLAD) and early language development in Mandarin-speaking children who received simultaneous bilateral cochlear implants (BICI) during the first year of cochlear implantation and compare the performance of the children who received BICI with those received unilateral cochlear implant (UCI).

**Methods:**

39 Mandarin-speaking children who received BICIs simultaneously and 36 children with UCIs were enrolled in this study. To access the EPLAD, the Infant-Toddler Meaningful Auditory Integration Scale (IT/MAIS) was conducted, and a subtest of the simplified short-form version of the Mandarin Communicative Development Inventory (SSF-MCDI) was used to evaluate the development of expressive and receptive vocabulary for the children at indicated time points after surgery.

**Results:**

In both the simultaneous BICI and UCI groups, we observed significantly increased scores of the SSF-MCDI and IT/MAIS 1 year after the surgery. There are indications of early advantages in children with BICI in IT/MAIS scores (at 1, 3, and 6 months after activation). For early development of language, a great difference between the expressive vocabulary scores and the receptive vocabulary scores was observed in both groups. We found there were not significant differences between the two groups on expressive or receptive vocabulary scores, the use of more differentiated measures might be required in future research. We further found that the development of the receptive or expressive vocabulary is dramatically correlated with the age at implantation and the total scores of IT/MAIS for children with simultaneous BICIs.

**Conclusion:**

These results may supplement the skills development of early language and auditory in Mandarin-native children with simultaneous CIs. It is obvious that children with normal hearing have mastery of receptive vocabulary before that of expressive vocabulary, which is the same as children with unilateral and bilateral CIs in this research. IT/MAIS total scores and age at CI were important factors for early language performance in children with simultaneous BICIs.

## Introduction

Cochlear implantation has become a promising method for improving hearing and speech performance in children with severe to profound sensorineural hearing loss (SNHL). UCIs provide access to sound for children with bilateral SNHL; however, when electrical input is provided on only one side, this type of asymmetric hearing may lead to abnormal cortical preference for the stimulated ear and auditory deprivation for the opposite ear over time. In order to achieve symmetric bilateral hearing (1, 2), an increasing number of scholars and experts (2, 3) have advocated simultaneous bilateral CI provision for pediatric candidates. Studies have shown that simultaneous BICI provides advantages, such as superior understanding of the speech in background noise, quality of life, and sound localization ability, compared with unilateral or sequential implantation (4–7). Several studies also showed that children subjected to the BICIs surgery perform better in EPLAD and language development in comparison to those with UCIs (8, 9).

For children speaking native Mandarin, a few studies have been conducted to investigate the EPLAD and the development of language in children subjected to the BICIs surgery. A study by Long et al. (10) found that the auditory preverbal skills of the BICI group were better than those of their unilateral CI peers during the 24 months of CI use. Another longitudinal study (11) explored the development of vocabulary in the children speaking native Mandarin after CIs and the faster growth of the receptive vocabulary was observed in the children with simultaneous BICIs compared to those with UCIs. In these studies, the limited sample sizes and inappropriate statistical analysis methods led to less rigorous research results. Generally, investigations evaluating the effects of early simultaneous bilateral stimulation on Mandarin-speaking children's prelingual auditory and language development over time are lacking.

Herein, we repeatedly evaluated the abilities of language and early auditory in the children speaking naïve mandarin after simultaneous BICI for 12 months after implantation, and their performance was compared with children with unilateral CIs. Furthermore, the fundamental and early auditory function's accumulation is an important process for the formation of language (12), we also hope to determine the factors influencing early language skills in the children speaking mandarin after simultaneous bilateral CIs.

## Materials and methods

### Participants

Children were enrolled by the Hearing Center of the Department of Otolaryngology, Head, and Neck Surgery of West China Hospital of Sichuan University from January 2018 to June 2020. The inclusion criteria were as follows: (1) all children presented with bilateral prelingual profound hearing loss, (2) received simultaneous BICIs or UCIs before 2 years of age, (3) for children with UCIs, had no contralateral HA, and (4) for all children, no other abnormalities related to development and malformation of inner ear were found. Additionally, all children in this study were hearing loss diagnosed before 6 months and the intervention for hearing aid was given before the CI treatment. Caregivers reported that their children used HA and CI more than 10 h per day, and all participants communicated with others in Mandarin in their daily lives. There are 39 children (11 girls and 28 boys) who were subjected to the simultaneous bilateral CIs were enrolled in the BICI group, and another 36 children (15 girls and 21 boys) who were subjected to the unilateral CIs and had matched gender and caregivers' education level were enrolled in the UCI group. Analysis of Mann–Whitney *U*-test was used to compare the two groups on these variables, there was no difference between two groups in Hearing Aid using time before CI, preoperative training time, postoperative training time, preoperative pure-tone audiometry average (PTA) and postoperative PTA, but it revealed a significant effect for age at implant (*p* = 0.019). Therefore, a linear mixed model that controlled for age at implant was employed. Furthermore, the parents of all children reported that the CIs were used throughout the day. Table 1 showed the children's demographic information. Audiological assessments of all children included otoacoustic emissions (OAEs), acoustic immittance, behavioural audiometric tests, and click and tone-burst elicited auditory brainstem response (ABR). There were five evaluation intervals: baseline and 1, 3, 6, and 12 months after CI activation. This study was approved by the Ethics Committee of West China Hospital of Sichuan University. All parents signed informed consent in this study.

**Table 1:** Patient demographics and audiological information.

	BICI group (*n* = 39)	UCI group (*n* = 36)	*Group- differences*
Gender, *n* (%)			
Female	11 (28.20)	15 (41.70)	*χ^2^*^a^* = 1*.*74 p = 0*.*187*
Male	28 (71.80)	21 (58.30)	* *
Age at CI (m), mean (SD)	14.5 (10.50)	19 (8.0)	***z*** **^b^** **= −2** **.** **343 *p *= 0** **.** **019**
HA using time Before CI (m)	5.03 (5.50)	5.5 (5.69)	*z *= −0.778 *p *= 0.437
Preoperative training time (m)	0.85 (2.34)	1.41 (3.20)	*z *= −0.305 *p *= 0.760
Postoperative training time (m)	10.28 (3.42)	9.46 (2.86)	*z *= −0.606 *p *= 0.545
Preoperative PTA (dB HL)	111.29 (6.96)	113.5 (6.14)	*z *= −0.954 *p *= 0.340
Postoperative PTA (dB HL)	38.43 (7.81)	35 (6.07)	*z *= −1.501 *p *= 0.133

CI, Cochlear Implant; HA, Hearing Aid; PTA, pure-tone audiometry average; BICI, simultaneous bilateral cochlear implant; UCI, unilateral cochlear implant; for BICI group, postoperative PTA is the mean value of two sides; and for UCI group, postoperative PTA average is the mean value of implanted side.

^a^ *χ*^2^ test.

^b^ Independent samples Mann–Whitney *U*-test; *p* = *p*-value.

* *p*-values below 0.05 (bold) indicate significant difference.

### Measurements

The Infant-Toddler Meaningful Auditory Integration Scale (IT/MAIS) is a structured interview with parents or caregivers to evaluate EPLAD in children before they are 3 years old (13) and was commonly employed for the assessment of children's auditory function before or after rehabilitation (14, 15). The Chinese version of the IT/MAIS was used to evaluate the EPLAD of participants in our study (16). Ten items which were divided into 3 main areas: vocalisation behaviour (items 1 and 2), sound detection (items 3–6), and sound discrimination and identification (items 7–10). Parents or caregivers were allowed 0–2 unanswered questions in one assessment.

Mandarin version of the MacArthur-Bates Communicative Development Inventory (CDI) (17) was adopted to assess the growth of the expressive and receptive vocabulary. It's a simplified short form (SSF) versions of CDI, which contained 50 items in each inventory (words and sentences (W&S) subtest; words and gestures (W&G) subtest) to evaluate vocabulary growth. The W&G subtest is suitable for children aged between 8 and 16 months, and the W&S subtest is suitable for children aged between 16 and 30 months. This study focused on the development of children 12 months after CI activation; hence, W&G Inventory was adopted. SSF versions of CDI has been not only used to assess early language development in developmentally normal children during routine health checks, but also to monitor a CI recipients' language progress after implantation (12). In our previous study, we found normalized receptive vocabulary growth rates during first 12 months after implantation in children implanted before 5 years of age are similar to those of normally hearing children under the age of 16 months. At 12 months after implantation, average performance on the receptive vocabulary score reaches ceiling. For expressive vocabulary score, it was less than those of normally hearing children. At 24 months after implantation, average performance on the expressive vocabulary score reaches ceiling, comparing with that of children 30 months of age with normal hearing.

### Data analysis

Data analysis and description were conducted using SPSS 26.0 (IBM, United States) and GraphPad Prism 8.0 (United States). Continuous variables are represented as the mean ± standard deviation (mean ± S.D.); categorical variables are expressed as the composition ratio.

In this study, based on the longitudinal study design with repeated measures, we made the comparison between BICI group and UCI group by a linear mixed model (LMM). This model is well-suited for longitudinal data, because they can account for variability between and across participants while being robust to missing data. In addition, it accounts for the correlation between the data at different time points (18). Our linear mixed models included group and follow-up intervals as fixed variables; evaluated the effect of time, group, and group-by-time interaction; and used random intercepts to account for participant-specific differences. Age at the time of CI was added to the model as a covariate. Thereafter, the mean differences between the children of the BICIs and UCIs groups at each follow-up interval were investigated using *post hoc* tests with Bonferroni adjustment. A third-order polynomial was used to create the curvature of the IT/MAIS scores using GraphPad Prism software. Spearman's product correlations were used to guide stepwise multiple regression analysis to assess the multivariate relationship between early language and auditory development. Statistical significance was defined as the *p*-value <0.05.

## Results

### IT/MAIS results in children with simultaneous BICIs versus UCIs

**Figure 1:**
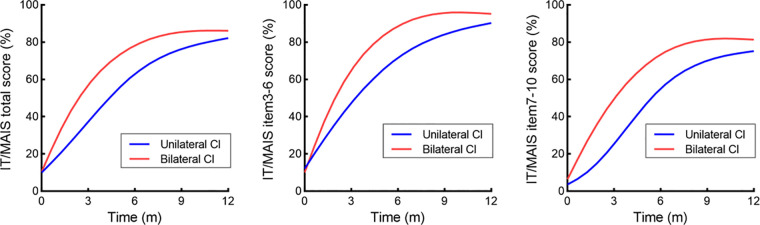
The trajectory of IT/MAIS scores using a third order polynomial.

Table 2 provides the results of the LMM analyse for IT/MAIS scores across the various follow-up intervals, and the model was adjusted for age at CI. The group × time interaction was significant for IT/MAIS total scores (*p* = 0.029), IT/MAIS 3–6 item scores (*p* = 0.014), and IT/MAIS 7–10 item scores (*p* = 0.024), indicating that the increase over time differed between the two groups. In addition, post-hoc tests with Bonferroni adjustment were carried out to compare scores of the children from BICIs and UCIs groups at indicated time points (Table 3). The total scores of IT/MAIS at 1 month were significantly different between the children of the simultaneous UCIs and BICIs groups, with a mean difference of 11.11 (*p* = 0.034). Similarly, the total scores of IT/MAIS at 3 (*p* = 0.007) and 6 months (*p* = 0.008) differed significantly. The scores of items 3–6 were not significantly different between groups over time, except at the 3-month (*p* = 0.002) and 6-month time points (*p* = 0.043). The differences of items 7–10 between the children who subjected to the simultaneous BICIs and UCIs at 3, 6, and 12 months were significant (*p* = 0.001, *p* = 0.018, and *p* = 0.001 respectively). However the differences of the total scores and the scores of item 3–6 at 12 months between the two groups were significant (*p* = 0.134 and *p* = 0.578 respectively). A third order polynomial by GraphPad Prism was used to visualise the developmental trajectory of IT/MAIS for the two groups. Figure 1A shows the IT/MAIS total score trajectory (we also display the trajectory of scores of items 3–6 and 7–10, Figures 1B,C). The scores in both the groups steadily increased over time.

**Table 2:** Summary statistics for the linear mixed-effects model for IT/MASI score, receptive vocabulary score and expressive vocabulary score.

	Numerator DF	Denominator DF	F	*p*-value
IT/MAIS total score				
Time	4	56.60	150.12	***<0*** **.** ***001*** *
Group	1	64.77	5.82	***0*** **.** ***019***
Time × group	4	56.59	2.93	***0*** **.** ***029***
Age at CI	1	56.28	0.13	*0*.*72*
IT/MAIS 3–6 items				
Time	4	31.22	144.83	***<0*** **.** ***001***
Group	1	26.94	3.12	*0*.*089*
Time × group	4	31.23	3.72	***0*** **.** ***014***
Age at CI	1	17.39	0.28	*0*.*606*
IT/MAIS 7–10 items				
Time	4	16.46	107.59	***<0*** **.** ***001***
Group	1	12.29	11.06	***0*** **.** ***006***
Time × group	4	16.44	3.74	***0*** **.** ***024***
Age at CI	1	9.51	.97	*0*.*350*
Receptive vocabulary score				
Time	4	2.93	110.38	***0*** **.** ***002***
Group	1	69.64	1.44	*0*.*234*
Time × group	4	2.93	3.31	*0*.*180*
Age at CI	1	94.82	1.13	*0*.*291*
Expressive vocabulary score				
Time	4	49.57	24.04	***<0*** **.** ***001***
Group	1	64.34	2.61	*0*.*111*
Time × group	4	49.47	0.77	*0*.*38*
Age at CI	1	49.33	1.53	*0*.*206*

* *p*-values below 0.05 (bold) indicate significant difference.

**Table 3:** Comparing BICI group to UCI group for each follow-up intervals.

Follow-up time	Contrast	Mean difference	Standard Error	*p-Value*
IT/MAIS total scores				
Baseline	Bilateral CI-Unilateral CI	2.65	4.75	*0*.*579*
1 month	Bilateral CI-Unilateral CI	11.11	5.14	***0*** **.** ***034*** *
3 months	Bilateral CI-Unilateral CI	17.40	6.25	***0*** **.** ***007***
6 months	Bilateral CI-Unilateral CI	16.21	5.91	***0*** **.** ***008***
12 months	Bilateral CI-Unilateral CI	8.95	5.91	*0*.*134*
IT/MAIS item 3–6 scores				
Baseline	Bilateral CI-Unilateral CI	0.22	2.45	*0*.*930*
1 month	Bilateral CI-Unilateral CI	2.67	2.73	*0*.*332*
3 months	Bilateral CI-Unilateral CI	8.74	2.67	***0*** **.** ***002***
6 months	Bilateral CI-Unilateral CI	5.77	2.72	***0*** **.** ***043***
12 months	Bilateral CI-Unilateral CI	1.92	3.44	*0*.*578*
IT/MAIS item 7–10 scores				
Baseline	Bilateral CI-Unilateral CI	1.14	2.71	*0*.*676*
1 month	Bilateral CI-Unilateral CI	5.56	2.61	*0*.*076*
3 months	Bilateral CI-Unilateral CI	10.33	2.99	***0*** **.** ***001***
6 months	Bilateral CI-Unilateral CI	7.52	2.81	***0*** **.** ***018***
12 months	Bilateral CI-Unilateral CI	14.80	4.19	***0*** **.** ***001***
Receptive vocabulary score				
Baseline	Bilateral CI-Unilateral CI	-4.34	5.60	*0*.*438*
1 month	Bilateral CI-Unilateral CI	2.78	6.15	*0*.*668*
3 months	Bilateral CI-Unilateral CI	7.12	6.80	*0*.*298*
6 months	Bilateral CI-Unilateral CI	20.78	7.21	***0*** **.** ***005***
12 months	Bilateral CI-Unilateral CI	3.58	7.76	*0*.*646*
Expressive vocabulary score				
Baseline	Bilateral CI-Unilateral CI	-0.20	0.68	*0*.*776*
1 month	Bilateral CI-Unilateral CI	0.49	0.80	*0*.*544*
3 months	Bilateral CI-Unilateral CI	1.27	2.05	*0*.*539*
6 months	Bilateral CI-Unilateral CI	7.30	5.27	*0*.*171*
12 months	Bilateral CI-Unilateral CI	16.70	10.08	*0*.*104*

Post hoc tests with Bonferroni adjustment was used to compare the scores between two groups.

* *p*-values below 0.05 (bold) indicate significant difference.

### The early language development in children with simultaneous BICIs versus UCIs

**Figure 2:**
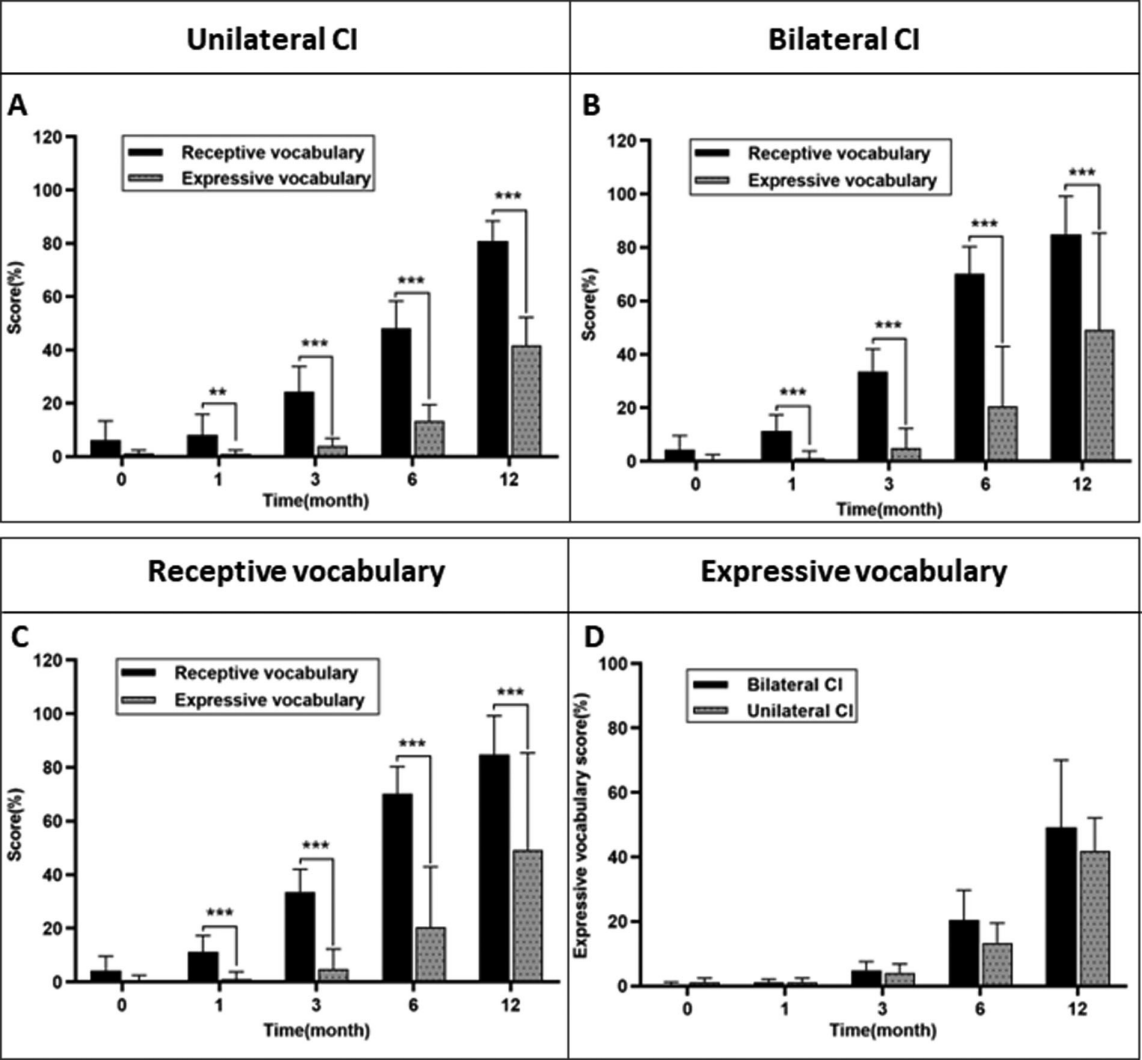
The mean scores of receptive and expressive vocabulary score in two groups. *p < 0.05, **p < 0.01.

Table 2 showed the LMM analyse for the scores of the expressive vocabulary and receptive vocabulary in the two groups of children at indicated time point. We did not observed the significant interaction between the group and time for either the scores of expressive vocabulary or the scores of receptive vocabulary, implying similar performance of these two groups over time. The main effect of time on receptive and expressive vocabulary scores was statistically significant (*p* = 0.002 and *p* < 0.001, respectively), suggesting that early language scores improved over time. Additionally, to investigate the scores of expressive and receptive vocabulary at differentially indicated time points, we conducted the Mann–Whitney *U* test. We found that the word reception scores at 1, 3, 6, and 12 months after CI activation were dramatically different from the scores of word expression in the two groups (Figures 2A,B, *p* < 0.01). To further compare the BICI and UCI groups, we conducted further analysis (Figures 2C,D). Figure 2C showed receptive vocabulary development for both groups to be very similar; a significant difference was observed after 6 months (*z* = −2.66, *p* = 0.008) and 12 months (*z* = −2.009, *p* = 0.045). However, no significant difference between the children from the BICIs group and UCIs group at 12 months after CI activation was observed. No significant difference of the word expression scores were found between the children of BICIs and UCIs groups at each follow-up time point.

### Factors associated with endpoint early language measures in children with simultaneous BICIs

**Figure 3:**
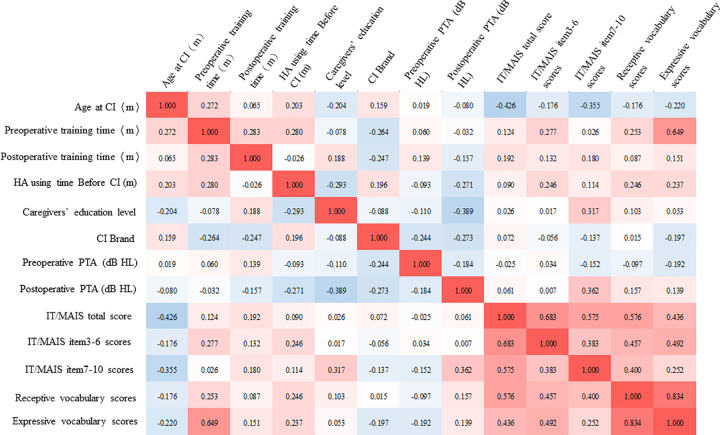
The correlational heat map shows between different predictive factors for children who received simultaneous bilateral CIs. The red filling represents the positive correlation, the blue filling represents the negative correlation, the depth of shade of the color represents the degree of correlation. Analyses were performed by the 2-tailed Spearman correlation analysis.

Several previous studies have demonstrated that better auditory development results in better vocabulary development (19). In our study, we also explored the correlation between EPLAD and the development of early language in children with simultaneous BICIs and found a high correlation between EPLAD scores and vocabulary (receptive and expressive) scores (Figure 3). Furthermore, the final independent variables entered into the stepwise regression included age at implantation, hearing aid use time before implantation, preoperative language training time, postoperative language training time, and preoperative and postoperative pure-tone audiometry averages. To assess the predictors associated with early language outcomes, we conducted the stepwise multivariate linear regression.

The regression yielded an *R*^2^ value of 0.849, which accounted for 84.9% of the variance in receptive vocabulary scores (*p* < 0.001). All independent variables, other than the IT/MAIS total scores, were excluded from the final regression model. For expressive vocabulary scores, 32% of the variation was attributed to the IT/MAIS total scores and age at implantation (Table 4).

**Table 4:** Results from stepwise multiple regression analysis, using dependent variables to predict simultaneous bilateral cochlear implant users’ receptive word and expressive scores.

Test	Predictor^a^	B	SE *B*	*β*	*t*	p*	Adj.R^2 b^
Receptive Word	IT/MAIS total Scores	1.029	0.111	0.927	9.251	**<0.001**	0.849
Expressive Word	IT/MAIS total Scores	0.813	0.375	0.462	2.166	**0.049**	0.320
	Age at CI (m)	4.220	1.908	0.472	2.212	**0.045**	

^a^ Predictor variables were selected using stepwise backward elimination.

^b^ Adj. *R*^2^, adjusted multiple correlation coefficient; SE B, SE of the regression coefficient.

* *p*-values below 0.05 (bold) indicate significant difference.

## Discussion

Although simultaneous bilateral CI has significantly increased worldwide over the past decades, due to the higher medical expenses or local health insurance policies, BICIs are not widely and commonly applied throughout China, and there are few longitudinal studies of early auditory development for the children speaking mandarin after simultaneous BICIs. In our study, we explored the early prelingual auditory and language development in these children and compare the performance with that of children with unilateral CIs. These results also allowed us to explore the effect of early prelingual auditory abilities on early language outcomes.

For children, elementary skills are EPLAD and necessary for the further advanced intelligence, cognition, communication, development of language and speech, and auditory function. Our results demonstrated that the children's EPLAD is significantly improved 1 year after CI activation. In addition, we found that the BICI group was more likely to have higher IT/MAIS scores than children with unilateral CIs at the 1-, 3-, and 6-month time points. Some previous studies have shown similar results; children with bilateral CIs displayed better early auditory skills than children implanted on one side. Long et al. used the Little EARS Auditory Questionnaire (LEAQ) to assess children's early auditory preverbal performance, which indicated that Mandarin-speaking children who received simultaneous BICI had better skills of the early preverbal auditory compared to those of UCI peers in the early stages after implantation (1, 3, and 6 months) (10). In their study, they used one-way analysis of variance (ANOVA) to compare the scores between the time points of measurements; in fact, it is inappropriate to use ANOVA to compare numerical variables between the time points of measurements, because the measurements are dependent on each other. In our study, we chose a linear mixed model to determine the difference in measurements between groups over time, which provided more appropriate information in terms of temporal changes because it works on variance changes in individual patients (20). We discovered that there was no significant difference in IT/MAIS total scores and items 3–6 scores between the children of the BICIs and UCIs groups after 12 months of CI usage, but the BICI group performed better than the UCI group in items 7–10 scores. As items 7–10 in the IT/MAIS evaluate the capacity of sound discrimination and identification, we conclude that children with bilateral CIs receive more benefits from sound discrimination and identification by 12 months of CI use, and more difficult assessment materials are required to assess their auditory skills in our future study.

During the first years of CI use, the two groups of receptive and expressive vocabulary developed in a pattern similar to that of children with normal hearing (Supplementary Table S2, data of children with normal hearing from Soli et al., 2012). The current study also showed that the children in two groups exhibited significantly high word reception scores in comparison with their word expression scores at each follow-up time point, indicating CI children are able to master the skill of word comprehension earlier than mastering the skill of word expression. Compared with children with normal hearing, children with CIs exhibited significantly higher receptive vocabulary scores and expressive vocabulary scores at each follow-up time point. One explanation is that the prelingual hearing loss children in this study receive the implant at an average chronological age of 14.5 months and 19 months in two groups, moreover they use hearing aids before implantation for different periods, thus they had a better cognitive level and learning ability than the normally developing children with the same hearing age.

We found that both groups showed steady improvements in general language skills (like the expressive and receptive vocabulary) in 1 year after the surgery. In comparison to the children of the BICIs group, the children of the UCIs group showed weak skills for expressive and receptive vocabulary, however, no significant difference was observed between them. Similar to our results, some other studies also did not show a significant beneficial effect of bilateral implantation on early language skills. Nittrouer and Chapman et al. evaluated receptive and expressive vocabulary on the 3.5-year-old children with BICIs, and found that the benefits from bilateral CI use are likely to be limited (21). A study for the outcomes of the language in children with BICIs after 3 years reported that no significant advantage for the skills of expressive and receptive vocabulary was found in the children with BICIs compared to those with UCIs by using the Reynell Developmental Language Scales (RDLS) (22). However, in earlier research performed on UCI users, there was evidence for significantly lower scores among the UCI users compared with their BICI peers. Julia Sarant et al. pointed out that the children of the BICIs group showed significantly fast development of language in comparison to those of the UCIs group (23). More importantly, this study was a cross-sectional study with a longer follow-up time (The average time of CI use were around 5 years). Accounting for these mixed results, due to the follow-up time was short, children could not get enough time to dramatically develop their language skills. Herein, we followed the development of receptive and expressive vocabulary for only 1 year after implantation, 12 months after BICIs activation are not enough for the obtaining of significant benefit.

Multiple regression analysis showed that higher IT/MAIS scores and earlier implantation were predictive of better language skills at 12 months after simultaneous implantation. It has been previously reported that better early prelingual auditory development leads to better development of vocabulary (7, 9, 24). Desjardin et al. showed that a child who performs well on sound perception will most likely be able to produce sounds in different words and sentences during daily conversational interactions (25). In addition, early access to auditory information given benefits to develop early language skills. In our samples, children with BICIs underwent surgery at a relatively young age (before the age of 16 months, *M* = 14.5 months), and we found that the age at CI is significantly associated with the ability of expressive vocabulary. Dettman et al. demonstrated that early implantation is significantly associated with the good performance of expressive vocabulary (26), they observed that in comparison to the children who received CIs at 4 years old, the children who received CIs younger than 12 months exhibited obviously high scores. In the study by Phan et al. (27) and Leigh et al. (28), they also found the age of most children implanted under 24 months performed near the ceiling level on early vocabulary skills by 1 year post-implantation. Interestingly, we did not find that HA use time before implantation correlated with early language development, although numerous studies observed the significant associations between the usage of HA before CIs and the better performance of vocabulary (29) and the better perception of sentence (30) in the children who speaking mandarin, possibly because the samples with bilateral CIs in our study were too small (only 39 children). It is necessary to enlarge the sample size in future research.

There are also several limitations in our study. First, no normal hearing group was available in this study for the comparison, so we don’t know whether the simultaneous BICIs results in similar development in early auditory and language abilities are existed in comparison to those normal peers. In previous studies, we use the term Normal Equivalent Age (NEA), which is synonymous with language age, to compare early language development scores in children with normal hearing and CI, the emphasis is placed on growth rates to avoid difficulties that can occur when comparing measures that span different age ranges (17). In future studies, we will further compare NEA between groups to illustrate the language development gap between children with CI and children with normal hearing. In this study, for children with unilateral CIs, early auditory development was significantly delayed in most follow-up intervals. Furthermore, as a future study, the children will be followed up for longer time in a prospective longitudinal design, the reason for extending the follow-up time is that living circumstances will change over time, and noisy classrooms and more advanced vocabulary might be confronted, which could reveal mixed results on language development in children subjected to BICIs.

In conclusion, children with simultaneous BICIs exhibited better performance on EPLAD compared with children with UCIs at some follow-up time points during 1 year after implantation. We also observed that language comprehension is necessary for the language expression, indicating that language comprehension should be given more attention in the early stages of rehabilitation. Furthermore, early prelingual auditory ability was significantly associated with the skills of early expressive and receptive vocabulary in the children after simultaneous BICIs.

## Data Availability

The original contributions presented in the study are included in the article/**Supplementary Material**, further inquiries can be directed to the corresponding author/s.
