# Supplementary material for: Early prelingual auditory and language development in children with simultaneous bilateral and unilateral cochlear implants
Source: Front Pediatr. 2022 Nov 3;10:999689. doi: 10.3389/fped.2022.999689 (PMC9669896; doi:10.3389/fped.2022.999689)
Supplement: Supplementary file 1 [file Table1.docx]

**Supplement Table1**  Model-based means for continuous outcomes after Bilateral CI and Unilateral CI

| **Outcome ^a^** | **Group** | **Baseline** | **1 mo** | **3 mo** | **6 mo** | **12 mo** |
| --- | --- | --- | --- | --- | --- | --- |
| IT/MAIS total score | Bilateral CI | 13.54  (7.56-19.52)^a^ | **27.82**  **(21.06-34.59)** | **55.23**  **(46.66-63.79)** | **78.81**  **(70.75-86.88)** | 88.35  (80.25-96.46) |
|  | Unilateral CI | 10.90  (3.66-18.13) | **16.71**  **(9.06-24.36)** | **37.82**  **(28.77-46.87)** | **62.60**  **(54.07-71.14)** | 79.40  (70.89-87.91) |
| Receptive vocabulary score | Bilateral CI | 1.78  (-4.75-8.30) | 9.94  (-0.26-20.14) | 31.73  (22.53-40.92) | **68.72**  **(58.47-78.97)** | 83.98  (71.62-96.34) |
|  | Unilateral CI | 6.12  (-2.80-15.05) | 7.16  (-4.30-18.62) | 24.61  (14.79-34.42) | **47.94**  **(37.98-57.90)** | 80.40  (71.31-89.49) |
| Expressive vocabulary score | Bilateral CI | 0.62  (-0.24-1.48) | 1.28  (0.25-2.31) | 4.91  (1.98-7.85) | 19.96  (12.04-27.89) | 55.90  (39.35-72.46) |
|  | Unilateral CI | 0.82  (-0.26-1.89) | 0.79  (-0.43-2.01) | 3.65  (0.79-6.50) | 12.66  (5.72-19.61) | 39.20  (27.55-50.87) |

1. All values are predicted means (95% confidence intervals) from linear mixed model for all patients, adjusted for age at CI.
2. ^a^ Values in parentheses are 95% confidence intervals.
3. ^c^ Main effect of group, *p* values below 0.05 (bold) indicate significant difference between groups.
