# Supplementary material for: Early prelingual auditory and language development in children with simultaneous bilateral and unilateral cochlear implants
Source: Front Pediatr. 2022 Nov 3;10:999689. doi: 10.3389/fped.2022.999689 (PMC9669896; doi:10.3389/fped.2022.999689)
Supplement: Supplementary file 2 [file Table2.docx]

**Supplement Table2** Normative scores of the SSF inventories（Soli et al. 2012）

| **Age (months)** | **Receptive vocabulary** | **Expressive vocabulary** |
| --- | --- | --- |
| 6 | 37.6% | 0.4% |
| 7 | 43.1% | 0.6% |
| 8 | 48.7% | 1.1% |
| 9 | 54.2% | 1.7% |
| 10 | 59.8% | 2.9% |
| 11 | 65.3% | 4.7% |
| 12 | 70.9% | 7.8% |
| 13 | 76.4% | 12.7% |
| 14 | 82.0% | 20.9% |
| 15 | 87.5% | 34.4% |
| 16 | 93.1% | 56.6% |
| 17 | 98.6% | 92.9% |
| 18 | 100.0% | 100.0% |
